# Fruiting Season Length Restricts Global Distribution of Female-Only Parental Care in Frugivorous Passerine Birds

**DOI:** 10.1371/journal.pone.0154871

**Published:** 2016-05-05

**Authors:** Sahas Barve, Frank A. La Sorte

**Affiliations:** 1 Department of Ecology and Evolutionary Biology, Cornell University, Ithaca, New York, United States of America; 2 Cornell Laboratory of Ornithology, Cornell University, Ithaca, New York, United States of America; Liverpool John Moores University, UNITED KINGDOM

## Abstract

Food availability is known to influence parental care and mating systems in passerine birds. Altricial chicks make uni-parental care particularly demanding for passerines and parental investment is known to increase with decreasing food availability. We expect this to limit uni-parental passerines to habitats with the most consistent food availability. In passerine birds, species having uni-parental care are primarily female-only parental care (female-only care) and most passerine birds with female-only care are frugivores. We predict that frugivorous passerines with female-only care should be restricted to the most stable habitats characterized by longer fruiting season length. At a global scale, female-only care frugivores were distributed in areas with significantly longer fruiting seasons than non-female-only care frugivores. Female-only care species richness had a stronger spatial relationship with longer fruiting season than non-female-only care species richness. Verifying the lack of a phylogenetic signal driving this pattern, our findings indicate that the geographic distribution of female-only care, a geographically and phylogenetically widespread parental care system, is restricted by an extrinsic factor: fruiting season length. This reinstates the importance of food availability on the evolution and maintenance of parental care systems in passerine birds.

## Introduction

Parental care is a crucial life-history strategy in birds. Bi-parental care, where the male and female raise chicks together is the most common strategy (*ca*. 81% of all species) [1]. However, other parental care systems such as multi-parental care and uni-parental care are also widespread [1]. Multi-parental care and uni-parental care are phylogenetically labile and have evolved independently in birds multiple times [1,2]. Mating systems in birds with altricial chicks, which depend on adults for food, are strongly associated with parental care [3]. All cooperative breeders show multi-parental care, bi-parental care is seen in socially monogamous and promiscuous species, while uni-parental care is almost exclusively found in polyandrous or polygynous species [4].

Parental care is especially important in passerine birds (songbirds) because chicks are altricial and undergo significant ontogenic development in the nest, making parental care particularly demanding [5]. In passerines both mating systems and modes of parental care are closely associated with food availability. For example, in acrocephaline (reed) warblers, cooperative breeding (multi-parental care) is seen in species occurring in habitats with low food supply. On the other hand, promiscuous and polygynous species (low male parental investment and uni-parental care, respectively) inhabit food rich habitats [6]. Globally, species that are cooperative breeders are most common in environments with high spatio-temporal resource unpredictability [7,8] but see [3] and male parental investment in numerous species increases with increasing environmental seasonality demonstrating the clear connection between food supply and seasonality of habitats [9,10]. An untested corollary is that uni-parental care should be restricted to areas with the most stable habitats with high resource certainty.

Uni-parental care in passerines is entirely female-only parental care (henceforth female-only care). Female-only care has multiple independent origins in several tropical frugivorous passerine families. These families represent two thirds of the known passerines that show female-only care (S1 Table). Frugivorous passerines also show a significantly higher proportion of female-only care, *ca*. 20% (109/561), than all other dietary guilds combined, *ca*. 2% (80/3700) [1]. Additionally, most transitions to female-only care in non-frugivorous passerines are associated with breeding in food rich environments and an increase in frugivory [1,9,11]. Frugivory, thus likely facilitates female-only care in passerines.

Birds that breed in the tropics, irrespective of their diet and mode of parental care, tend to have smaller clutch sizes and multiple broods in a year [12]. Additionally, frugivores have further smaller clutch sizes than omnivores or granivores [13]. Despite being nutritionally inferior to insects, fleshy fruits are abundant and easier to acquire [14]. Frugivory, as opposed to insectivory, thus makes provisioning for adults and/or young easier. Therefore, frugivory further reduces parental investment already offset through small clutches at every breeding attempt [15,16]. Multiple small broods and reduced parental investment due to frugivory might offset the costs of a lack of male parental care. Multiple broods and hence long breeding season is however only likely in areas with long periods of food (fruit) availability for frugivores.

The tropics are characterized by limited seasonality, a lack of sharp fluctuations in food availability, and limited temporal variation in the abundance of food resources (fruits and insects) with availability remaining consistent for long periods of the year [17–19]. Fruiting season length varies globally. It is shortest in the high latitudes and is longest in the tropics. Ting et al. [20] performed a global analysis using 48 studies to understand environmental predictors of fruiting season length. Evapotranspiration, the quantity of water that is actually removed from a surface due to the processes of evaporation and transpiration, precipitation and temperature were all associated with fruiting season length, out of which evapotranspiration showed the highest positive correlation and was identified as the best predictor [20]. Equatorial tropical forests with high evapotranspiration globally have the longest fruiting seasons and thus represent aseasonal environments with consistent food availability [17]. Lengthy periods of food availability might present ideal conditions for sustaining multiple small broods of frugivores. The relationship between fruiting season and evapotranspiration thus allows us to investigate the relationship between food availability and the spatial distribution of modes of parental care in frugivores. Due to the complete lack of male parental investment among passerine frugivores, we predict that female-only care should be restricted to equatorial areas with significantly longer fruiting seasons than frugivores with bi-parental care.

In birds, variation in food availability due to seasonality is an important determinant of parental investment [7,9]. We provide the first formal test of the role that low seasonality and stable food availability plays in allowing female-only care to evolve and persist in a comparative study of all the world’s frugivorous passerine birds. Here we define frugivores after Kissling et al. [21] as birds with a high proportion of fleshy fruits in their diet. Using the robustly established correlation between evapotranspiration and the length of the fruiting season [20], we array all frugivorous passerines along a gradient of evapotranspiration (i.e., fruiting season length). We demonstrate that female-only care species are distributed in a small subset of the global tropics. We confirm the strong geographic association between fruiting season length and global distribution of female-only care within this taxonomically diverse dietary guild and verify the lack of a strong phylogenetic signal in our data. We substantiate how stable food availability might drive the spatial distribution of an entire parental care system in birds.

## Materials and Methods

### Datasets

We compiled published lists of passerine frugivorous birds from [21] and female-only care passerine species from [1]. We acquired breeding range maps for the world’s bird species from BirdLife International and NatureServe [22]. We standardized taxonomies using BirdLife International [23]. This procedure resulted in a total of 561 frugivore passerine species for analysis (S1 Table). Of these, 109 were identified as female-only care species (S1 Table).

### Analyses

We converted the breeding range map polygons to equal-area hexagon cell with a spatial resolution of 12,452 km^2^ using a icosahedral discrete global grid system defined by a Fuller icosahedral projection using an aperture 4 hexagon partition method [24,25]. Evapotranspiration was estimated globally for the combined period 2000 to 2013 using MODIS16A3 (spatial resolution = 1 km^2^) [26]. We averaged the gridded evapotranspiration values within the larger hexagon cells based on the location of the 1-km^2^ cell centres.

We used a permutation procedure to determine how the geographic distributions of frugivore passerine species with and without female-only care differed spatially. First, we calculated the species richness of female-only care species and non-female-only care species across hexagon cells. We then calculated the proportion of female-only care species within each cell. To determine which cells had unusually high or low percentages of female-only care species, we shuffled without replacement the female-only care classification among the 561 species. We then recalculated the percentage of female-only care and non-female-only care species per cell. We repeated this procedure 99 times, each time calculating the difference between these percentages for each cell. We then calculated the average difference in the percentages for each cell, whose values had a range from 100 to -100 (higher to lower than excepted proportion of female-only care species, respectively).

In addition, we used kernel density analysis [27] to determine if female-only care and non-female-only care species richness had different global associations with evapotranspiration. This approach summarized the spatial relationship between the number of species and the evapotranspiration value estimated for each hexagon cell. We used the contour levels of the kernel density surface to document the primary associations for each class of species and to determine how this relationship differed between the two classes. We chose a kernel density approach because of the strong non-linear relationship between species richness and evapotranspiration when examined in a spatially explicit fashion across the globe.

To ensure that our findings were not driven by a few closely related taxonomic groups. We constructed a phylogeny of 403/561 species of passerines frugivores with genetic data using a posterior set of pruned trees from Jetz *et al*. (2012) [28]. We carried out a phylogenetic logistic regression between a binary variable (female-only care = 1, other = 0) and the mean of evapotranspiration within the geographic range each species.

We used R, version 3.2.4 to conduct all analyses [29]. We implemented the kernel density analysis using the kde function in the ks package [30]. The phylogenetic logistic regression analysis was implemented using the packages phylolm [31] and ape [32].

## Results

**Fig 1:**
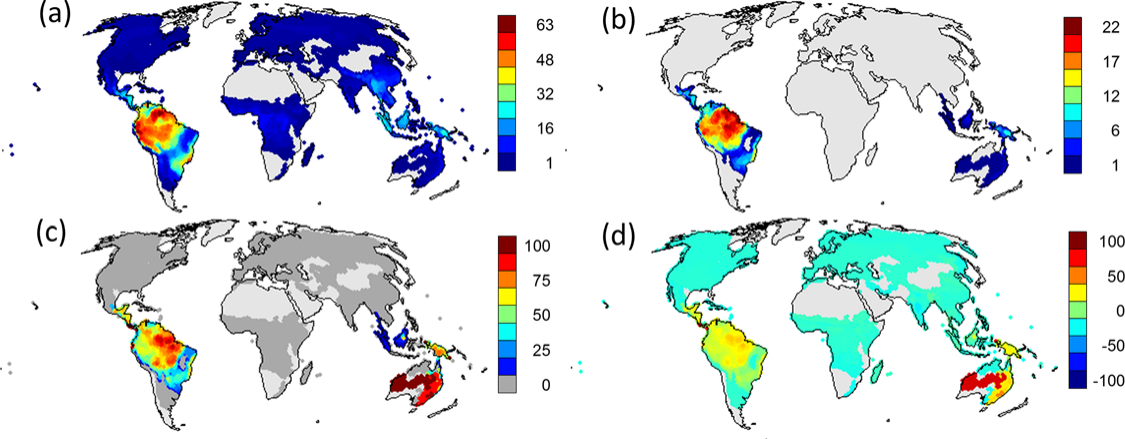
Global Distribution of Frugivory and Parental Care Mode. Global species richness of (a) frugivore passerine bird species (*n* = 561) and (b) frugivore passerine bird species that display female-only parental care (*n* = 109). (c) The proportion of frugivore passerine bird species that display female-only care and (d) the results of a permutation test estimating the likelihood of these proportions occurring by chance alone. Values range from 100 (red), higher than expected, to -100 (blue), lower than expected. Information in each map is summarized within equal-area cells of a global icosahedron (spatial resolution = 12,452 km^2^) and the map projection is Mollweide.

The greatest concentration of the 561 frugivorous passerine species occurred in the tropics (Fig 1A). As predicted, frugivorous passerine species with female-only care were limited to the equatorial regions of tropical Central and South America, Southeast Asia and Australia with the curious exception of Africa (Fig 1B). The greatest proportion of frugivorous passerine species with female-only care occurred within Australia, South America, and New Guinea (Fig 1C). When considering the likelihood of female-only care occurring by chance alone among the distribution of frugivore passerine species (Fig 1A and 1B), higher than expected percentage occurred within Australia, South America, and New Guinea, and lower than expected percentage occurred throughout regions where non-female-only care frugivore passerines occurred (Fig 1D).

**Fig 2:**
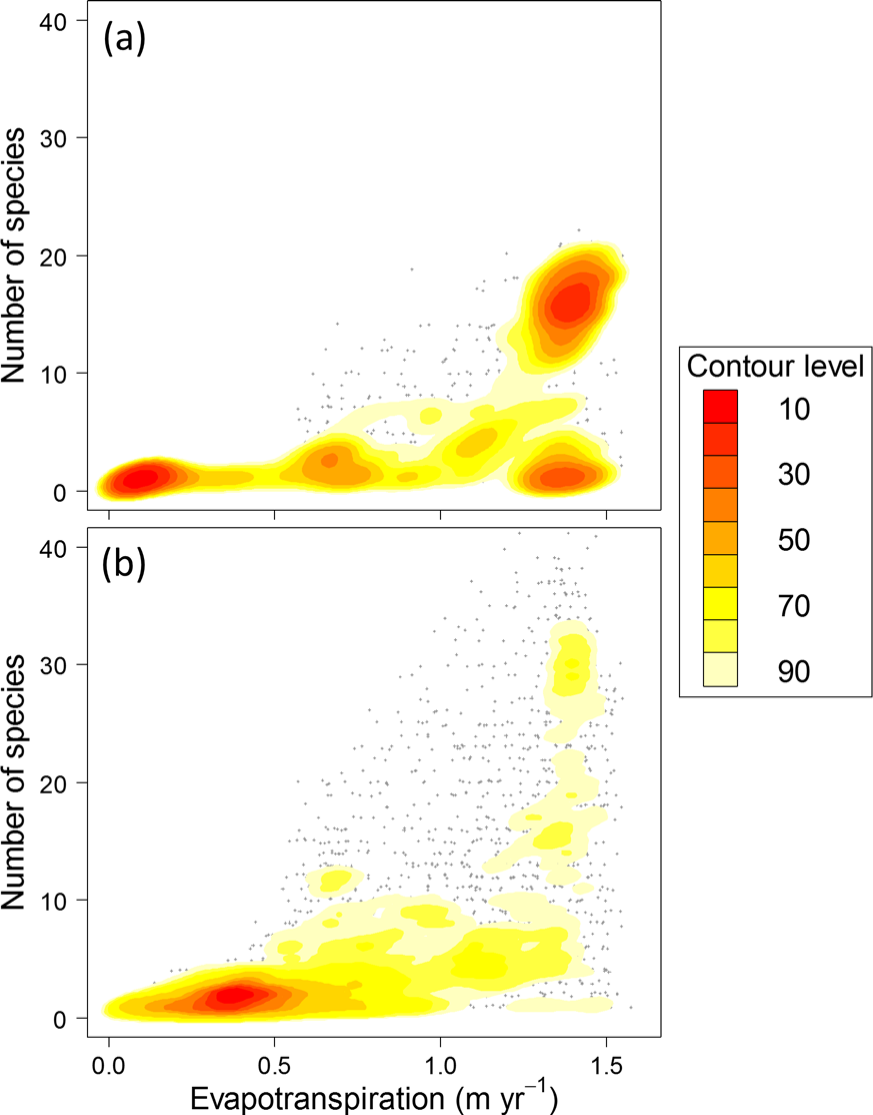
**Kernel Density Analysis Of Species Richness For Frugivore Parental Care Type** (a) frugivore passerine bird species that display female-only parental care (*n* = 109) and (b) frugivore passerine bird species that display other forms of parental care (*n* = 452) as a function of evapotranspiration within equal-area hexagon cells of a global icosahedron (spatial resolution = 12,452 km^2^). The filled contours are kernel density estimates at 10% intervals from 10% to 90%. The intervals correspond to the upper percentages of the highest density regions. The grey points are hexagon cells whose values occur outside the 90% contour interval.

Among frugivore passerines, female-only care species occurred in geographic regions with significantly higher evapotranspiration relative to non-female-only care species (*t* = 2.95, df = 190, *P* = 0.004). When the spatial relationship between species richness and evapotranspiration was examined, female-only care species presented contrasting associations from species that display other forms of parental care (Fig 2). For female-only care species, cells containing low numbers of species occurred in regions characterized by low evapotranspiration, and cells containing high numbers of species only occurred in regions with high evapotranspiration (Fig 2A). For species that display other forms of parental care, the dominant association was characterized by cells containing low numbers of species occurring in regions with low evapotranspiration (Fig 2B). Thus, when examined in a spatially explicit fashion, only female-only care species displayed a strong association between high species numbers and high values of evapotranspiration. The phylogenetic logistic regression analysis verified the lack of phylogenetic signal in the association between average evapotranspiration and parental care type (*α* = 0.014, *P* = 0.234).

## Discussion

Our spatially explicit analysis shows that most frugivorous passerines that have female-only care are concentrated in, and restricted to, a small global area of the tropics with significantly higher evapotranspiration and thus longer fruiting seasons relative to frugivore passerines showing other modes of parental care. Female-only care has evolved independently in frugivorous passerines in both the oscine and sub-oscine sub-orders of the Passeriformes [1]. Yet, the same extrinsic factor of food availability, namely fruiting season length, significantly explains the global geographic distribution of female-only care in over a hundred species of birds.

Frugivorous birds have evolved several life history and morphological characters as adaptation to a frugivorous diet [33, 34]. Fruits are an abundant but nutritionally inferior resource compared to insects [14,35]. Growth rates in nestlings of frugivores have been shown to be slower than in insectivorous birds [14,36] as fruits have lower protein content [33]. This lengthens the nestling period increasing the chance of predation. Long nestling periods are expected to drive species to have smaller clutch sizes, which reduces the overall level of activity at the nest [14, 37]. Tropical birds globally have small clutch sizes [16,38] and frugivores have smaller clutch sizes than omnivores and granivores [13]. However nesting seasons in the tropics are long and hence tropical frugivores have multiple clutches of a single or a few eggs [39]. The increased risk of predation due to brightly coloured males tending to nests has been a long-standing hypothesis to explain the evolution of uni-parental care in several frugivorous passerine birds [40,41]. However, the lack of male investment in parental care places a significant cost on the female. The distribution of female only care in frugivorous passerines is largely limited to areas with protracted fruiting seasons. This pattern supports our prediction that, with the lack of male parental care and multiple small broods with long developmental periods driven by frugivory and predation, female only care will only occur in areas with the longest periods of food availability for frugivores. Thus our findings provide a testable spatial prediction complimenting the hypothesis that the loss of male parental care is due to increased predation in the tropics.

In the equatorial tropics, although fruiting seasons are long, fruit resources are often patchily distributed over a large area. Frugivores are known to track these locally concentrated but temporally transient food resources [42] over large spatial scales and thus have large home ranges [43, 44], which makes defending a territory difficult. In fact, tropical evergreen forest frugivorous birds are often less territorial than temperate forest species [45]. The lack of territoriality, and multiple broods across the year may allow females to move nesting locations for successive broods close to existing spatially accessible fruit resources [46].

Frugivorous bird families that display female-only care, such as the cotingas (Cotingidae) and the birds-of-paradise (Paradisaeidae), represent the best-known examples of polygyny and extreme plumage dimorphism in birds. Hence polygyny (a mating system) and female-only care (a parental care system) have evolved independently multiple times in frugivorous passerines. Several hypotheses have been proposed as mechanisms for the evolution of this extreme plumage dimorphism [47, 48]. The spatio-temporal nature of fruit resource dispersion, make it difficult to defend fruit resources. The inability of males to guard food resources has been famously hypothesized for the evolution of lek systems in these frugivorous passerines [49–52]. We speculate that the evolution of female-only care in these same frugivorous passerines due to spatio-temporally patchy food resources that are difficult to defend but multiple small clutches and lengthy periods food availability that can be spatially tracked might have further increased importance of sexual selection in these species. Mating systems and modes of parental care are intricately intertwined in passerines [4]. We do not aim to explain the processes that led to the magnificent sexual dimorphism in these species. On the contrary, our findings present an additional alternative hypothesis that the viability of female-only care in passerine frugivores of the equatorial tropics alleviating the need for male parental investment might have had a strong influence on the evolution of the remarkable dimorphism and ploygyny seen in many female-only care species.

All frugivores in the equatorial tropics do not show female-only care. Similarly, not all female-only care species show extreme sexual dimorphism. We therefore acknowledge that ecology and evolutionary history have played an important role in the evolution of parental care in these clades [53]. We constrain our analyses to frugivorous passerines because, with altricial chicks and a globally predictable food resource, they best satisfy the requirements to test our hypothesis.

Female-only care is found in frugivores in all tropical areas with the exception of Africa. However, Africa has very few specialized frugivorous passerines. This absence is attributed to the dearth of plant species in the families Lauraceae and Palmae, which have large fleshy fruits. These plant families support numerous specialized frugivores in the Neotropics and Australasia and are thought to have experienced substantial extinctions in Africa [54]. Australia has the highest proportion of passerine frugivores that have female-only care (Fig 1D). This pattern is driven by two species of female-only care bowerbirds (western (*Chlamydera guttata*) and spotted bowerbirds (*Chlamydera maculate*)), which make up a large proportion of the passerine frugivores despite the low evapotranspiration levels within the region. Our goal in this study was not to explore the evolutionary origins and diversification of female-only care species, but we do confirm that our results are not driven by closely related species suggesting a strong role for geography in defining the spatial distribution of female-only care.

In non-frugivorous passerines, female-only care is found in species that breed in exceptionally food-rich habitats and have elevated levels of frugivory than their congeners [11, 18, 55]. Female-only care in passerines thus is linked with increased frugivory and or breeding in environments with high food availability. This demonstrates a global association between food availability and uni-parental care systems in passerines.

## Conclusion

Passerines show a diverse array of parental care systems, and seasonality and food availability drive parental investment in passerines due to altricial chicks [6, 7, 19]. Low seasonality and long periods of fruit availability might have facilitated the evolution and persistence of female-only care in tropical frugivorous passerines, and fruiting season length might in turn have restricted the global distribution of this trait. Thus, climate and geography have been important not only in the distribution of species but also in the distribution of unique reproductive strategies such as female-only care.

## Supporting Information

S1 Table Frugivore Passerines of the World The 561 frugivore passerine bird species considered in the analysis. (PDF)

S2 Table The passerine taxonomic families considered in the analysis. The passerine taxonomic families considered in the analysis and number of species within each parental care category.
(PDF)
