# Supplementary material for: Fruiting Season Length Restricts Global Distribution of Female-Only Parental Care in Frugivorous Passerine Birds
Source: PLoS One. 2016 May 5;11(5):e0154871. doi: 10.1371/journal.pone.0154871 (PMC4858211; doi:10.1371/journal.pone.0154871)
Supplement: S1 Table — (PDF) [file pone.0154871.s001.pdf]

SUPPORTING INFORMATION

Fruiting season length restricts global distribution of female-only parental care in frugivorous passerine birds

Sahas Barve and Frank A. La Sorte

Appendix S1 The 561 frugivore passerine bird species considered in the analysis and the 109 species that display female-only parental care (1) and the 452 species that display other forms of parental care (0).

Species	Family	Parental care
Bombycilla cedrorum	Bombycillidae	0
Bombycilla garrulus	Bombycillidae	0
Bombycilla japonica	Bombycillidae	0
Dulus dominicus	Bombycillidae	0
Phainopepla nitens	Bombycillidae	0
Phainoptila melanoxantha	Bombycillidae	0
Ptilogonys caudatus	Bombycillidae	0
Ptilogonys cinereus	Bombycillidae	0
Coracina larvata	Campephagidae	0
Coracina lineata	Campephagidae	0
Coracina montana	Campephagidae	0
Lalage moesta	Campephagidae	0
Loboparadisea sericea	Campephagidae	1
Caryothraustes canadensis	Cardinalidae	0
Chlorothraupis stolzmanni	Cardinalidae	0
Periporphyrus erythromelas	Cardinalidae	0
Piranga bidentata	Cardinalidae	0
Rhodothraupis celaeno	Cardinalidae	0
Saltator orenocensis	Cardinalidae	0
Cnemophilus loriae	Cnemophilidae	1
Cnemophilus macgregorii	Cnemophilidae	1
Coracina schisticeps	Corvidae	0
Corvus florensis	Corvidae	0
Corvus fuscicapillus	Corvidae	0
Corvus hawaiiensis	Corvidae	0
Corvus jamaicensis	Corvidae	0
Corvus meeki	Corvidae	0
Corvus tristis	Corvidae	0
Corvus typicus	Corvidae	0

Corvus unicolor	Corvidae	0
Corvus validus	Corvidae	0
Cyanocorax affinis	Corvidae	0
Cyanocorax caeruleus	Corvidae	0
Platysmurus leucopterus	Corvidae	0
Urocissa caerulea	Corvidae	0
Ampelioides tschudii	Cotingidae	0
Ampelion rubrocristatus	Cotingidae	0
Ampelion rufaxilla	Cotingidae	0
Calyptura cristata	Cotingidae	0
Carpodectes antoniae	Cotingidae	1
Carpodectes hopkei	Cotingidae	1
Carpodectes nitidus	Cotingidae	1
Carpornis cucullata	Cotingidae	0
Carpornis melanocephala	Cotingidae	0
Cephalopterus glabricollis	Cotingidae	1
Cephalopterus ornatus	Cotingidae	1
Cephalopterus penduliger	Cotingidae	1
Conioptilon mcilhennyi	Cotingidae	0
Cotinga amabilis	Cotingidae	1
Cotinga cayana	Cotingidae	1
Cotinga cotinga	Cotingidae	1
Cotinga maculata	Cotingidae	1
Cotinga maynana	Cotingidae	0
Cotinga nattererii	Cotingidae	1
Cotinga ridgwayi	Cotingidae	1
Doliornis remseni	Cotingidae	0
Doliornis sclateri	Cotingidae	0
Gymnoderus foetidus	Cotingidae	1
Haematoderus militaris	Cotingidae	1
Lipaugus fuscocinereus	Cotingidae	1
Lipaugus lanioides	Cotingidae	1
Lipaugus streptophorus	Cotingidae	1
Lipaugus unirufus	Cotingidae	1
Lipaugus uropygialis	Cotingidae	1
Lipaugus vociferans	Cotingidae	1
Perissocephalus tricolor	Cotingidae	1
Phibalura flavirostris	Cotingidae	0
Phoenicircus carnifex	Cotingidae	1
Phoenicircus nigricollis	Cotingidae	1
Pipreola arcuata	Cotingidae	0
Pipreola aureopectus	Cotingidae	0
Pipreola chlorolepidota	Cotingidae	0

Pipreola formosa	Cotingidae	0
Pipreola frontalis	Cotingidae	0
Pipreola intermedia	Cotingidae	0
Pipreola jucunda	Cotingidae	0
Pipreola lubomirskii	Cotingidae	0
Pipreola pulchra	Cotingidae	0
Pipreola riefferii	Cotingidae	0
Pipreola whitelyi	Cotingidae	0
Porphyrolaema porphyrolaema	Cotingidae	0
Procnias albus	Cotingidae	0
Procnias averano	Cotingidae	1
Procnias nudicollis	Cotingidae	1
Procnias tricarunculatus	Cotingidae	1
Pyroderus scutatus	Cotingidae	1
Querula purpurata	Cotingidae	0
Rupicola peruvianus	Cotingidae	1
Rupicola rupicola	Cotingidae	1
Snowornis cryptolophus	Cotingidae	0
Snowornis subalaris	Cotingidae	0
Tijuca atra	Cotingidae	0
Tijuca condita	Cotingidae	0
Xipholena atropurpurea	Cotingidae	1
Xipholena lamellipennis	Cotingidae	1
Xipholena punicea	Cotingidae	1
Zaratornis stresemanni	Cotingidae	0
Chlorospingus flavovirens	Emberizidae	0
Chlorospingus semifuscus	Emberizidae	0
Calyptomena hosii	Eurylaimidae	1
Calyptomena viridis	Eurylaimidae	1
Calyptomena whiteheadi	Eurylaimidae	1
Pseudocalyptomena graueri	Eurylaimidae	0
Chlorophonia callophrys	Fringillidae	0
Chlorophonia cyanea	Fringillidae	0
Chlorophonia flavirostris	Fringillidae	0
Chlorophonia occipitalis	Fringillidae	0
Chlorophonia pyrrhophrys	Fringillidae	0
Euphonia affinis	Fringillidae	0
Euphonia anneae	Fringillidae	0
Euphonia cayennensis	Fringillidae	0
Euphonia chalybea	Fringillidae	0
Euphonia chlorotica	Fringillidae	0
Euphonia chrysopasta	Fringillidae	0

Euphonia concinna	Fringillidae	0
Euphonia cyanocephala	Fringillidae	0
Euphonia elegantissima	Fringillidae	0
Euphonia finschi	Fringillidae	0
Euphonia fulvicrissa	Fringillidae	0
Euphonia gouldi	Fringillidae	0
Euphonia hirundinacea	Fringillidae	0
Euphonia imitans	Fringillidae	0
Euphonia jamaica	Fringillidae	0
Euphonia laniirostris	Fringillidae	0
Euphonia luteicapilla	Fringillidae	0
Euphonia mesochrysa	Fringillidae	0
Euphonia minuta	Fringillidae	0
Euphonia musica	Fringillidae	0
Euphonia pectoralis	Fringillidae	0
Euphonia plumbea	Fringillidae	0
Euphonia rufiventris	Fringillidae	0
Euphonia saturata	Fringillidae	0
Euphonia trinitatis	Fringillidae	0
Euphonia violacea	Fringillidae	0
Euphonia xanthogaster	Fringillidae	0
Heterospingus rubrifrons	Fringillidae	0
Psittirostra psittacea	Fringillidae	0
Hypocolius ampelinus	Hypocoliidae	0
Cacicus cela	Icteridae	1
Cacicus chrysonotus	Icteridae	0
Cacicus chrysopterus	Icteridae	0
Curaeus forbesi	Icteridae	0
Gymnomystax mexicanus	Icteridae	0
Hypopyrrhus pyrohypogaster	Icteridae	0
Icterus laudabilis	Icteridae	0
Psarocolius bifasciatus	Icteridae	1
Psarocolius cassini	Icteridae	1
Psarocolius decumanus	Icteridae	1
Psarocolius montezuma	Icteridae	1
Psarocolius viridis	Icteridae	1
Chloropsis aurifrons	Irenidae	0
Melanocharis arfakiana	Melanocharitidae	0
Melanocharis crassirostris	Melanocharitidae	0
Melanocharis longicauda	Melanocharitidae	0
Melanocharis nigra	Melanocharitidae	0
Melanocharis striativentris	Melanocharitidae	0
Melanocharis versteri	Melanocharitidae	0

Lichenostomus flavescens	Meliphagidae	0
Macgregoria pulchra	Meliphagidae	0
Meliphaga montana	Meliphagidae	0
Melipotes ater	Meliphagidae	0
Melipotes fumigatus	Meliphagidae	0
Melipotes gymnops	Meliphagidae	0
Oreornis chrysogenys	Meliphagidae	0
Myophonus blighi	Muscicapidae	0
Myophonus melanurus	Muscicapidae	0
Turdus albocinctus	Muscicapidae	0
Dicaeum aeruginosum	Nectariniidae	0
Dicaeum agile	Nectariniidae	0
Dicaeum aureolimbatum	Nectariniidae	0
Dicaeum haematostictum	Nectariniidae	0
Dicaeum monticolum	Nectariniidae	0
Dicaeum nitidum	Nectariniidae	0
Oriolus albiloris	Oriolidae	0
Oriolus auratus	Oriolidae	0
Oriolus bouroensis	Oriolidae	0
Oriolus chinensis	Oriolidae	0
Oriolus chlorocephalus	Oriolidae	0
Oriolus crassirostris	Oriolidae	0
Oriolus cruentus	Oriolidae	0
Oriolus flavocinctus	Oriolidae	0
Oriolus forsteni	Oriolidae	0
Oriolus hosii	Oriolidae	0
Oriolus isabellae	Oriolidae	0
Oriolus melanotis	Oriolidae	0
Oriolus monacha	Oriolidae	0
Oriolus oriolus	Oriolidae	0
Oriolus percivali	Oriolidae	0
Oriolus phaeochromus	Oriolidae	0
Oriolus sagittatus	Oriolidae	0
Oriolus steerii	Oriolidae	0
Oriolus szalayi	Oriolidae	0
Oriolus xanthonotus	Oriolidae	0
Pitohui dichrous	Oriolidae	0
Sphecotheres hypoleucus	Oriolidae	0
Sphecotheres vieilloti	Oriolidae	0
Sphecotheres viridis	Oriolidae	0
Astrapia mayeri	Paradisaeidae	1
Astrapia nigra	Paradisaeidae	1
Astrapia rothschildi	Paradisaeidae	1

Astrapia splendidissima	Paradisaeidae	1
Astrapia stephaniae	Paradisaeidae	0
Cicinnurus magnificus	Paradisaeidae	1
Cicinnurus regius	Paradisaeidae	1
Cicinnurus respublica	Paradisaeidae	1
Epimachus bruijnii	Paradisaeidae	1
Epimachus fastuosus	Paradisaeidae	1
Lycocorax pyrrhopterus	Paradisaeidae	0
Manucodia ater	Paradisaeidae	0
Manucodia chalybatus	Paradisaeidae	0
Manucodia comrii	Paradisaeidae	0
Manucodia jobiensis	Paradisaeidae	0
Manucodia keraudrenii	Paradisaeidae	0
Paradigalla brevicauda	Paradisaeidae	0
Paradigalla carunculata	Paradisaeidae	0
Paradisaea apoda	Paradisaeidae	1
Paradisaea decora	Paradisaeidae	1
Paradisaea guilielmi	Paradisaeidae	1
Paradisaea minor	Paradisaeidae	0
Paradisaea raggiana	Paradisaeidae	1
Paradisaea rubra	Paradisaeidae	1
Paradisaea rudolphi	Paradisaeidae	1
Parotia carolae	Paradisaeidae	1
Parotia helenae	Paradisaeidae	0
Parotia lawesii	Paradisaeidae	1
Parotia sefilata	Paradisaeidae	1
Parotia wahnesi	Paradisaeidae	1
Pteridophora alberti	Paradisaeidae	1
Ptiloris intercedens	Paradisaeidae	0
Ptiloris magnificus	Paradisaeidae	1
Seleucidis melanoleucus	Paradisaeidae	1
Semioptera wallacii	Paradisaeidae	0
Oreocharis arfaki	Paramythiidae	0
Paramythia montium	Paramythiidae	0
Erythrura papuana	Passeridae	0
Antilophia galeata	Pipridae	0
Chiroxiphia boliviana	Pipridae	1
Chiroxiphia caudata	Pipridae	0
Chiroxiphia lanceolata	Pipridae	1
Chiroxiphia linearis	Pipridae	1
Chiroxiphia pareola	Pipridae	1
Corapipo gutturalis	Pipridae	1
Corapipo leucorrhoa	Pipridae	1

Heterocercus aurantiivertex	Pipridae	0
Heterocercus flavivertex	Pipridae	0
Heterocercus linteatus	Pipridae	0
Ilicura militaris	Pipridae	1
Lepidothrix coeruleocapilla	Pipridae	0
Lepidothrix coronata	Pipridae	0
Lepidothrix iris	Pipridae	0
Lepidothrix isidorei	Pipridae	0
Lepidothrix nattereri	Pipridae	0
Lepidothrix serena	Pipridae	0
Lepidothrix suavissima	Pipridae	0
Lepidothrix vilasboasi	Pipridae	0
Machaeropterus deliciosus	Pipridae	1
Machaeropterus pyrocephalus	Pipridae	1
Machaeropterus regulus	Pipridae	1
Manacus aurantiacus	Pipridae	1
Manacus candei	Pipridae	1
Manacus manacus	Pipridae	1
Manacus vitellinus	Pipridae	0
Masius chrysopterus	Pipridae	1
Pipra aureola	Pipridae	1
Pipra chloromeros	Pipridae	1
Pipra cornuta	Pipridae	0
Pipra erythrocephala	Pipridae	1
Pipra fasciicauda	Pipridae	1
Pipra filicauda	Pipridae	1
Pipra mentalis	Pipridae	1
Pipra pipra	Pipridae	1
Pipra rubrocapilla	Pipridae	1
Xenopipo atronitens	Pipridae	1
Xenopipo flavicapilla	Pipridae	1
Xenopipo holochlora	Pipridae	0
Xenopipo unicolor	Pipridae	1
Xenopipo uniformis	Pipridae	1
Ailuroedus buccoides	Ptilonorhynchidae	0
Ailuroedus crassirostris	Ptilonorhynchidae	0
Ailuroedus melanotis	Ptilonorhynchidae	0
Amblyornis flavifrons	Ptilonorhynchidae	1
Amblyornis inornata	Ptilonorhynchidae	1
Amblyornis macgregoriae	Ptilonorhynchidae	1
Amblyornis subalaris	Ptilonorhynchidae	1
Archboldia papuensis	Ptilonorhynchidae	1

Chlamydera cerviniventris	Ptilonorhynchidae	1
Chlamydera guttata	Ptilonorhynchidae	1
Chlamydera lauterbachi	Ptilonorhynchidae	1
Chlamydera maculata	Ptilonorhynchidae	1
Chlamydera nuchalis	Ptilonorhynchidae	0
Prionodura newtoniana	Ptilonorhynchidae	1
Ptilonorhynchus violaceus	Ptilonorhynchidae	1
Scenopoeetes dentirostris	Ptilonorhynchidae	1
Sericulus aureus	Ptilonorhynchidae	0
Sericulus bakeri	Ptilonorhynchidae	0
Sericulus chrysocephalus	Ptilonorhynchidae	0
Alophoixus flaveolus	Pycnonotidae	0
Baeopogon clamans	Pycnonotidae	0
Baeopogon indicator	Pycnonotidae	0
Calyptocichla serina	Pycnonotidae	0
Chlorocichla flavicollis	Pycnonotidae	0
Chlorocichla laetissima	Pycnonotidae	0
Chlorocichla prigoginei	Pycnonotidae	0
Chlorocichla simplex	Pycnonotidae	0
Hemixos castanonotus	Pycnonotidae	0
Hypsipetes borbonicus	Pycnonotidae	0
Hypsipetes leucocephalus	Pycnonotidae	0
Hypsipetes madagascariensis	Pycnonotidae	0
Hypsipetes mcclellandii	Pycnonotidae	0
Hypsipetes nicobariensis	Pycnonotidae	0
Hypsipetes olivaceus	Pycnonotidae	0
Hypsipetes parvirostris	Pycnonotidae	0
Hypsipetes thompsoni	Pycnonotidae	0
Hypsipetes virescens	Pycnonotidae	0
Iole olivacea	Pycnonotidae	0
Iole propinqua	Pycnonotidae	0
Iole virescens	Pycnonotidae	0
Ixonotus guttatus	Pycnonotidae	0
Ixos everetti	Pycnonotidae	0
Ixos malaccensis	Pycnonotidae	0
Ixos palawanensis	Pycnonotidae	0
Ixos philippinus	Pycnonotidae	0
Ixos rufigularis	Pycnonotidae	0
Ixos siquijorensis	Pycnonotidae	0
Phyllastrephus strepitans	Pycnonotidae	0
Pycnonotus atriceps	Pycnonotidae	0
Pycnonotus aurigaster	Pycnonotidae	0
Pycnonotus bimaculatus	Pycnonotidae	0

Pycnonotus blanfordi	Pycnonotidae	0
Pycnonotus brunneus	Pycnonotidae	0
Pycnonotus cyaniventris	Pycnonotidae	0
Pycnonotus erythropthalmos	Pycnonotidae	0
Pycnonotus eutilotus	Pycnonotidae	0
Pycnonotus finlaysoni	Pycnonotidae	0
Pycnonotus flavescens	Pycnonotidae	0
Pycnonotus goiavier	Pycnonotidae	0
Pycnonotus leucogrammicus	Pycnonotidae	0
Pycnonotus melanicterus	Pycnonotidae	0
Pycnonotus melanoleucos	Pycnonotidae	0
Pycnonotus nieuwenhuisii	Pycnonotidae	0
Pycnonotus penicillatus	Pycnonotidae	0
Pycnonotus plumosus	Pycnonotidae	0
Pycnonotus priocephalus	Pycnonotidae	0
Pycnonotus simplex	Pycnonotidae	0
Pycnonotus sinensis	Pycnonotidae	0
Pycnonotus squamatus	Pycnonotidae	0
Pycnonotus striatus	Pycnonotidae	0
Pycnonotus taivanus	Pycnonotidae	0
Pycnonotus tympanistrigus	Pycnonotidae	0
Pycnonotus urostictus	Pycnonotidae	0
Pycnonotus xantholaemus	Pycnonotidae	0
Pycnonotus xanthorrhous	Pycnonotidae	0
Spizixos semitorques	Pycnonotidae	0
Tricholestes criniger	Pycnonotidae	0
Rhagologus leucostigma	Rhagologidae	0
Aplonis atrifusca	Sturnidae	0
Aplonis cantoroides	Sturnidae	0
Aplonis crassa	Sturnidae	0
Aplonis mavornata	Sturnidae	0
Aplonis opaca	Sturnidae	0
Aplonis pelzelni	Sturnidae	0
Aplonis santovestris	Sturnidae	0
Aplonis striata	Sturnidae	0
Aplonis tabuensis	Sturnidae	0
Aplonis zelandica	Sturnidae	0
Enodes erythrophris	Sturnidae	0
Gracula ptilogenys	Sturnidae	0
Margarops fuscus	Sturnidae	0
Sarcops calvus	Sturnidae	0
Scissirostrum dubium	Sturnidae	0
Streptocitta albertinae	Sturnidae	0

Parophasma galinieri	Sylviidae	0
Anisognathus igniventris	Thraupidae	0
Anisognathus lacrymosus	Thraupidae	0
Anisognathus melanogenys	Thraupidae	0
Anisognathus notabilis	Thraupidae	0
Anisognathus somptuosus	Thraupidae	0
Bangsia arcaei	Thraupidae	0
Bangsia aureocincta	Thraupidae	0
Bangsia edwardsi	Thraupidae	0
Bangsia melanochlamys	Thraupidae	0
Bangsia rothschildi	Thraupidae	0
Buthraupis aureodorsalis	Thraupidae	0
Buthraupis eximia	Thraupidae	0
Buthraupis montana	Thraupidae	0
Buthraupis wetmorei	Thraupidae	0
Calochaetes coccineus	Thraupidae	0
Chlorochrysa calliparaea	Thraupidae	0
Chlorophanes spiza	Thraupidae	0
Chlorornis riefferii	Thraupidae	0
Chrysothlypis chrysomelas	Thraupidae	0
Chrysothlypis salmoni	Thraupidae	0
Cissopis leverianus	Thraupidae	0
Conirostrum leucogenys	Thraupidae	0
Dacnis albiventris	Thraupidae	0
Dacnis berlepschi	Thraupidae	0
Dacnis cayana	Thraupidae	0
Dacnis flaviventer	Thraupidae	0
Dacnis hartlaubi	Thraupidae	0
Dacnis lineata	Thraupidae	0
Dacnis nigripes	Thraupidae	0
Dacnis venusta	Thraupidae	0
Dacnis viguieri	Thraupidae	0
Delothraupis castaneoventris	Thraupidae	0
Diglossa glauca	Thraupidae	0
Diglossa indigotica	Thraupidae	0
Dubusia taeniata	Thraupidae	0
Heterospingus xanthopygius	Thraupidae	0
Iridophanes pulcherrimus	Thraupidae	0
Iridosornis jelskii	Thraupidae	0
Iridosornis porphyrocephalus	Thraupidae	0
Iridosornis reinhardti	Thraupidae	0
Iridosornis rufivertex	Thraupidae	0

Lamprospiza melanoleuca	Thraupidae	0
Loxipasser anoxanthus	Thraupidae	0
Mitrospingus cassinii	Thraupidae	0
Mitrospingus oleagineus	Thraupidae	0
Pipraeidea melanonota	Thraupidae	0
Ramphocelus bresilius	Thraupidae	0
Ramphocelus carbo	Thraupidae	0
Ramphocelus costaricensis	Thraupidae	0
Ramphocelus dimidiatus	Thraupidae	0
Ramphocelus flammigerus	Thraupidae	0
Ramphocelus melanogaster	Thraupidae	0
Ramphocelus nigrogularis	Thraupidae	0
Ramphocelus passerinii	Thraupidae	0
Ramphocelus sanguinolentus	Thraupidae	0
Schistochlamys melanopis	Thraupidae	0
Schistochlamys ruficapillus	Thraupidae	0
Sericossypha albocristata	Thraupidae	0
Spindalis dominicensis	Thraupidae	0
Spindalis nigricephala	Thraupidae	0
Spindalis portoricensis	Thraupidae	0
Spindalis zena	Thraupidae	0
Stephanophorus diadematus	Thraupidae	0
Tangara argyrofenges	Thraupidae	0
Tangara arthus	Thraupidae	0
Tangara brasiliensis	Thraupidae	0
Tangara cabanisi	Thraupidae	0
Tangara callophrys	Thraupidae	0
Tangara cayana	Thraupidae	0
Tangara chilensis	Thraupidae	0
Tangara chrysotis	Thraupidae	0
Tangara cucullata	Thraupidae	0
Tangara cyanicollis	Thraupidae	0
Tangara cyanocephala	Thraupidae	0
Tangara cyanoptera	Thraupidae	0
Tangara cyanotis	Thraupidae	0
Tangara cyanoventris	Thraupidae	0
Tangara desmaresti	Thraupidae	0
Tangara dowii	Thraupidae	0
Tangara fastuosa	Thraupidae	0
Tangara florida	Thraupidae	0
Tangara fucosa	Thraupidae	0
Tangara guttata	Thraupidae	0
Tangara gyrola	Thraupidae	0

Tangara heinei	Thraupidae	0
Tangara icterocephala	Thraupidae	0
Tangara inornata	Thraupidae	0
Tangara johannae	Thraupidae	0
Tangara larvata	Thraupidae	0
Tangara lavinia	Thraupidae	0
Tangara mexicana	Thraupidae	0
Tangara meyerdeschauenseei	Thraupidae	0
Tangara nigrocincta	Thraupidae	0
Tangara nigroviridis	Thraupidae	0
Tangara palmeri	Thraupidae	0
Tangara parzudakii	Thraupidae	0
Tangara peruviana	Thraupidae	0
Tangara phillipsi	Thraupidae	0
Tangara preciosa	Thraupidae	0
Tangara punctata	Thraupidae	0
Tangara ruficervix	Thraupidae	0
Tangara rufigenis	Thraupidae	0
Tangara rufigula	Thraupidae	0
Tangara schrankii	Thraupidae	0
Tangara seledon	Thraupidae	0
Tangara varia	Thraupidae	0
Tangara vassorii	Thraupidae	0
Tangara velia	Thraupidae	0
Tangara viridicollis	Thraupidae	0
Tangara vitriolina	Thraupidae	0
Tangara xanthocephala	Thraupidae	0
Tangara xanthogastra	Thraupidae	0
Tersina viridis	Thraupidae	0
Thraupis abbas	Thraupidae	0
Thraupis bonariensis	Thraupidae	0
Thraupis cyanocephala	Thraupidae	0
Thraupis cyanoptera	Thraupidae	0
Thraupis episcopus	Thraupidae	0
Thraupis glaucocolpa	Thraupidae	0
Thraupis ornata	Thraupidae	0
Thraupis palmarum	Thraupidae	0
Thraupis sayaca	Thraupidae	0
Wetmorethraupis sterrhopteron	Thraupidae	0
Garrulax affinis	Timalidae	0
Garrulax bieti	Timalidae	0
Garrulax caerulatus	Timalidae	0

Garrulax henrici	Timalidae	0
Garrulax jerdoni	Timalidae	0
Garrulax morrisonianus	Timalidae	0
Garrulax sannio	Timalidae	0
Liocichla omeiensis	Timalidae	0
Liocichla phoenicea	Timalidae	0
Liocichla steerii	Timalidae	0
Lioptilus nigricapillus	Timalidae	0
Iodopleura fusca	Tityridae	0
Iodopleura isabellae	Tityridae	0
Iodopleura pipra	Tityridae	0
Laniisoma buckleyi	Tityridae	0
Laniocera rufescens	Tityridae	1
Cataponera turdoides	Turdidae	0
Chlamydochaera jefferyi	Turdidae	0
Cochoa azurea	Turdidae	0
Cochoa beccarii	Turdidae	0
Cochoa purpurea	Turdidae	0
Cochoa viridis	Turdidae	0
Myadestes coloratus	Turdidae	0
Myadestes genibarbis	Turdidae	0
Myadestes lanaiensis	Turdidae	0
Myadestes melanops	Turdidae	0
Myadestes myadestinus	Turdidae	0
Myadestes obscurus	Turdidae	0
Myadestes palmeri	Turdidae	0
Myadestes unicolor	Turdidae	0
Turdus amaurochalinus	Turdidae	0
Turdus assimilis	Turdidae	0
Turdus flavipes	Turdidae	0
Turdus fulviventris	Turdidae	0
Turdus fuscater	Turdidae	0
Turdus jamaicensis	Turdidae	0
Turdus leucops	Turdidae	0
Turdus ludoviciae	Turdidae	0
Turdus maculirostris	Turdidae	0
Turdus migratorius	Turdidae	0
Turdus nigriceps	Turdidae	0
Turdus nudigenis	Turdidae	0
Turdus obsoletus	Turdidae	0
Turdus pallidus	Turdidae	0
Turdus pelios	Turdidae	0
Turdus pilaris	Turdidae	0

Turdus plebejus	Turdidae	0
Turdus serranus	Turdidae	0
Turdus unicolor	Turdidae	0
Zoothera peronii	Turdidae	0
Legatus leucophaius	Tyrannidae	0
Mionectes macconnelli	Tyrannidae	1
Mionectes oleagineus	Tyrannidae	1
Mionectes olivaceus	Tyrannidae	0
Mionectes rufiventris	Tyrannidae	1
Mionectes striaticollis	Tyrannidae	1
Myiodynastes luteiventris	Tyrannidae	0
Tyrannulus elatus	Tyrannidae	0
Zimmerius bolivianus	Tyrannidae	0
Zimmerius chrysops	Tyrannidae	0
Zimmerius cinereicapilla	Tyrannidae	0
Zimmerius gracilipes	Tyrannidae	0
Zimmerius improbus	Tyrannidae	0
Zimmerius vilissimus	Tyrannidae	0
Zimmerius viridiflavus	Tyrannidae	0
Apalopteron familiare	Zosteropidae	0
