# Supplementary material for: Fruiting Season Length Restricts Global Distribution of Female-Only Parental Care in Frugivorous Passerine Birds
Source: PLoS One. 2016 May 5;11(5):e0154871. doi: 10.1371/journal.pone.0154871 (PMC4858211; doi:10.1371/journal.pone.0154871)
Supplement: S2 Table — The passerine taxonomic families considered in the analysis and number of species within each parental care category. (PDF) [file pone.0154871.s002.pdf]

**SUPPORTING INFORMATION**

**Fruiting season length restricts global distribution of female-only parental care in frugivorous passerine birds**

Sahas Barve and Frank A. La Sorte

**Appendix S2** The passerine taxonomic families considered in the analysis. We report the proportion of species in the family showing frugivory (Proportion of frugivory in family), the number of species within each family considered as female-only care (Female-only) and classified as having other forms of parental care (Other).

| Family | Proportion of frugivory in family | Other | Female-only |
|---|---|---|---|
| Bombycillidae | 0.89 | 8 | 0 |
| Campephagidae | 0.06 | 4 | 1 |
| Cardinalidae | 0.14 | 6 | 0 |
| Cnemophilidae | 0.67 | 0 | 2 |
| Corvidae | 0.11 | 14 | 0 |
| Cotingidae | 0.87 | 30 | 32 |
| Emberizidae | 0.01 | 2 | 0 |
| Eurylaimidae | 0.27 | 1 | 3 |
| Fringillidae | 0.24 | 34 | 0 |
| Hypocoliidae | 1.00 | 1 | 0 |
| Icteridae | 0.11 | 6 | 6 |
| Irenidae | 0.50 | 1 | 0 |
| Melanocharitidae | 0.60 | 6 | 0 |
| Meliphagidae | 0.04 | 7 | 0 |
| Muscicapidae | 0.03 | 3 | 0 |
| Nectariniidae | 0.05 | 6 | 0 |
| Oriolidae | 0.80 | 24 | 0 |
| Paradisaeidae | 0.83 | 13 | 22 |
| Paramythiidae | 1.00 | 2 | 0 |
| Passeridae | 0.03 | 1 | 0 |
| Pipridae | 0.72 | 16 | 26 |
| Ptilonorhynchidae | 0.95 | 7 | 12 |
| Pycnonotidae | 0.42 | 58 | 0 |

| | | | |
|---|---|---|---|
| Rhagologidae | 1.00 | 1 | 0 |
| Sturnidae | 0.14 | 16 | 0 |
| Sylviidae | 0.00 | 1 | 0 |
| Thraupidae | 0.43 | 123 | 0 |
| Timalidae | 0.04 | 11 | 0 |
| Tityridae | 0.17 | 4 | 1 |
| Turdidae | 0.10 | 34 | 0 |
| Tyrannidae | 0.03 | 11 | 4 |
| Zosteropidae | 0.01 | 1 | 0 |
